# Analysis of Electrical Brain Waves in Neurotoxicology: Gamma-Hydroxybutyrate

**DOI:** 10.2174/157015911795017209

**Published:** 2011-03

**Authors:** Z.K Binienda, M.A Beaudoin, B.T Thorn, S.F Ali

**Affiliations:** 1 Division of Neurotoxicology; FDA/NCTR, Jefferson, AR, USA; 2 Z-Tech, Co.; FDA/NCTR, Jefferson, AR, USA; 3 Division of Personalized Nutrition and Medicine; FDA/NCTR, Jefferson, AR, USA

**Keywords:** Domoic acid, ibogaine, cocaine, gamma hydroxybutyrate, cerebral cortex, electrocorticogram, power spectra.

## Abstract

Advances in computer technology have allowed quantification of the electroencephalogram (EEG) and expansion of quantitative EEG (qEEG) analysis in neurophysiology, as well as clinical neurology, with great success. Among the variety of techniques in this field, frequency (spectral) analysis using Fast Fourier Transforms (FFT) provides a sensitive tool for time-course studies of different compounds acting on particular neurotransmitter systems. Studies presented here include Electrocorticogram (ECoG) analysis following exposure to a glutamic acid analogue - domoic acid (DOM), psychoactive indole alkaloid - ibogaine, as well as cocaine and gamma-hydroxybutyrate (GHB). The ECoG was recorded in conscious rats *via* a tether and swivel system. The EEG signal frequency analysis revealed an association between slow-wave EEG activity delta and theta and the type of behavioral seizures following DOM administration. Analyses of power spectra obtained in rats exposed to cocaine alone or after pretreatment with ibogaine indicated the contribution of the serotonergic system in ibogaine mediated response to cocaine (increased power in alpha_1_ band). Ibogaine also lowered the threshold for cocaine-induced electrographic seizures (increased power in the low-frequency bands, delta and theta). Daily intraperitoneal administration of cocaine for two weeks was associated with a reduction in slow-wave ECoG activity 24 hrs following the last injection when compared with controls. Similar decreased cortical activity in low-frequency bands observed in chronic cocaine users has been associated with reduced metabolic activity in the frontal cortex. The FFT analyses of power spectra relative to baseline indicated a significant energy increase over all except beta_2_ frequency bands following exposure to 400 and 800 mg/kg GHB. The EEG alterations detected in rats following exposure to GHB resemble absence seizures observed in human *petit mal* epilepsy. Spectral analysis of the EEG signals combined with behavioral observations may prove to be a useful approach in studying chronic exposure to drugs of abuse and treatment of drug dependence.

## INTRODUCTION

Electroencephalography and neuroimaging are major techniques in brain investigation. Electrocerebral activity represents local action potentials and widespread excitatory and inhibitory postsynaptic potentials recorded as the EEG. ECoG records an average of synchronous, widespread post-synaptic potentials arising in vertically oriented pyramidal cells of the upper layers of the cerebral cortex [1]. Advances in computer technology have allowed quantification of the EEG and expansion of quantitative EEG (qEEG) analysis in neurophysiology, as well as clinical neurology, with a great success. Among the variety of techniques in this field, the computer assisted frequency (spectral) analysis (FFT) of the EEG signals provides a sensitive tool for time-course studies of different compounds acting on particular neurotransmitter systems. Frequency data are often analyzed as the power spectrum, measured as total power in microvolts-squared divided by frequency (µV^2^/Hz) and separated into low (delta, theta) or high (alpha, beta) frequency bands.

## ANIMALS, INSTRUMENTATION, DATA ANALYSIS

Adult, male Sprague-Dawley rats of the Charles River cesarean delivered (CD) strain were used in the study.Animals were kept under controlled environmental conditions (temperature 22^o^C, relative humidity 45-55%, 12-h light/dark cycle) and housed individually with NIH-41 Irradiated Rodent Diet (Harlan Teklad, Madison, WI) chow and tap water supplied *ad libitum.* Bipolar stainless steel electrodes were implanted 3 mm laterally from the sagittal fissure, 1 and 4 mm posterior to the bregma. They were referenced to a ground electrode placed in the dorsal neck. The ECoG was recorded *via* a tether and swivel system at least one week after implantation. During recording, the animals remained in a microdialysis bowl placed inside a Faraday cage. Amplified signals were rectified to pass frequencies of 1-40 Hz and processed with Lab View software (National Instruments, Austin, TX). The power spectra obtained by use of FFT were divided into 1.25-4.5 Hz (delta), 4.75—6.75 (theta), 7.00-9.50 Hz (alpha_1_), 9.75-12.50 Hz (alpha_2_), 12.75-18.5 Hz (beta_1_), and 18.75-35.00 Hz (beta_2_) frequency bands [2]. Following the intraperitoneal (i.p.) saline injection and 30-min baseline recording, rats were treated i.p. with compounds of interest and recording continued for the next 60 -120 min.

All animal procedures were approved by the Institutional Animal Care and Use Committee (IACUC) of the NCTR and conducted in full accordance with the PHS policy on humane care and use of laboratory animals and the NIH guide for the care and use of laboratory animals.

In order to analyze data statistically, each power measurement during treatment was divided by the median power in the reference run (baseline) in order to place all animals on the same scale despite factors that made the absolute power different between animals. Data were subsequently normalized using a log transformation. Power difference between a 5-min interval and the baseline level 30-min period was tested against zero using the Student’s* t*-test. Values of p < 0.05 were considered statistically significant.

## DOMOIC ACID (DOM) STUDY

DOM, a contaminant of some seafood was identified as the culprit in mussel poisoning. Intoxication was marked by neurological symptoms including seizures and behavioral symptoms. An increase in the release of glutamate and a decrease of the release of GABA are consistent with excitotoxic mechanism of DOM toxicity [3]. Results of the brain wave analysis demonstrated that FFT can simplify the conversion of raw EEG signals into a numerically useful form. An FFT analysis of the ECoG recorded from rats before and after i.p. dosing with DOM revealed high voltage activity (electrographic seizures) not accompanied initially by any behavioral seizures. The high dose (4.4 mg/kg) DOM group ECoG was elevated beginning 30 min postinjection, whereas in the low dose (2.2 mg/kg) group became elevated only after 110 min (Fig. (**1**)). Further division of the ECoG data into frequency bands indicated that the initial power increase was mainly at the lower frequencies delta and theta. Statistically significant elevations in delta and theta waves were observed both sooner and at a lower dose than size-related behaviors [4].

## IBOGAINE AND COCAINE STUDIES

A psychoactive indole alkaloid, ibogaine, has been evaluated in several laboratories for its properties as an anti-addictive drug. However in animal studies, exposure to ibogaine has been associated with neurotoxic side effects. In our study, spectral analysis of the ECoG in rats injected with cocaine HCl alone (20 mg/kg, i.p. ), or pretreated with ibogaine (50 mg/kg), followed one hour later by cocaine, revealed that administration of cocaine alone was associated only with a significant power increase in the alpha_1_ frequency band during the first 30 min postinjection. The alpha_1_ increase was maintained throughout recording when cocaine was injected after ibogaine pretreatment. The spectral pattern obtained after ibogaine/cocaine treatment showed increased power in the low frequency bands and enhancement of power in the alpha_1_ band, indicating the contribution of the serotonergic system in the ibogaine-mediated response to cocaine. The enhancement of power observed in low frequency bands after the combined treatment with ibogaine/cocaine suggests that ibogaine at high doses decreases the threshold for cocaine-induced seizures [5].

The FFT analysis of ECoG recorded in rats injected with 15 mg/kg cocaine HCl i.p. for two weeks revealed a significant decrease in slow-wave frequency bands (delta, theta). Data were consistent with reported findings in cocaine-dependent human subjects where repeated exposure to cocaine resulted in a decrease in slow-wave brain activity showing usefulness of the animal model developed in our lab in the study of human cocaine dependence [6].

## GHB STUDY

GHB, a short chain fatty acid and a GABA derivative, has been characterized as an inhibitory neurotransmitter in the brain and neuromodulator of the dopaminergic, opioid and GABA-ergic neurotransmitter systems [7, 8]. While approved by the US FDA for the treatment of narcolepsy, the abuse of GHB as a psychotropic drug became a world-wide problem by the late 90’s. Known to recreational users as *G, gib, soap, salty water or nitro*, exposure to GHB induces short term amnesia, increases libido and euphoria. The disinhibition of sexual behavior produced by GHB has also given it the moniker of a *date-rape drug* because of its abuse in sexual assaults.

Quantitative autoradiography and imaging techniques have allowed the detection of high-affinity GHB receptor sites, particularly dense in the neuronal cells of forebrain region such as the striatum - including the nucleus accumbens, hippocampus, and thalamus [9]. A cloning technique allowed identification of a GHB specific receptor that has been postulated to exist in the sites matching the regions of GABA_B._ [10]. The interaction with GABA_B_ receptors leads to an inhibition in dopamine (DA) release and alters of the EEG profiles, as well as behavioral effects such as sedation, and decreases motor activity [11].

As was expected due to the ability of GHB to block the firing of DA neurons, microdialysis sampling revealed that acute GHB administration in awake rats leads to a decrease in striatal DA compared with baseline levels [12, 13]. In turn, the pharmacokinetic/pharmacodynamic model allowed detection of an IC_50 _increase in the GHB inhibition of the DA synthesis rate following chronic exposure to GHB, indicating decreased DA sensitivity toward the GHB inhibitory action [13].

The effect of GHB on electroencephalographic signals has been investigated in animal models and human [14-18]. While acute exposure to exogenous GHB in rats at low doses (50-100 mg/kg) induced immobility and sleep, a hypersynchronous electrographic seizures resembling generalized nonconvulsive epilepsy were observed following GHB at doses higher than 200 mg/kg [15]. These EEG changes were blocked by GABA_B _receptor antagonists [16]. Chronic exposure to GHB in the rat led to a reduced sleep period reflected by the EEG, and indicated end-organ sensitivity, i.e., tolerance to the hypnotic action of GHB [19].

In our study, following a saline injection and 30 min recording of baseline ECoG, rats were treated i.p. with GHB at 100, 200, 400, and 800 mg/kg and recording continued for the next 60 min. The real-time ECoG recording indicated a dose response in bilateral spike-and-wave discharges consistent with electrographic seizures. These 5-7 sec spikes, initially observed within 7-10 min following 200 mg/kg GHB, were accompanied by a desynchronized ECoG, remaining progressively longer and lasting throughout the 60-min recording period in 400 and 800 mg/kg GHB treated rats. The alterations in ECoG activity were associated with behavioral sedation (“freezing”) characterized by immobility and staring. The FFT analyses of power spectra relative to baseline indicated a significant energy increase over all, except beta_2_, frequency bands following 400 and 800 mg/kg GHB (Fig. (**2**)). The significant power increases after 400 mg/kg GHB were observed within approximately 30 min compared to 10-15 min after 800 mg/kg. The EEG alterations detected in rats following exposure to GHB resemble absence seizures observed in human *petit mal* epilepsy.

## CONCLUSIONS

The FFT mathematical conversion applied to EEG signals takes into account both frequency and amplitude of the signal within defined powers (frequency bands). The frequency analysis revealed differences between a particular class of drugs allowing “fingerprinting” of hallucinogenic and non-hallucinogenic amphetamine derivatives. While an increase in alpha_1_ power correlating with a serotonin increase were observed in hallucinogenic actions, the spectral patterns following exposure to non-hallucinogens were characterized by a decrease in the alpha_2_ and delta band [2]. 

Spectral analysis applied in our studies allowed association of low frequency power bands delta and theta activity with the early development of convulsant activity within the limbic system following exposure to DOM. The EEG events preceded motoric seizures. The FFT –processed ECoG successfully measured increases in delta and theta that were below the threshold of voltage or duration required to induce seizure-related behaviors. The enhancement of spectral power observed in low frequency bands after the ibogaine/cocaine treatment indicating lower threshold for seizures was somehow contradictory to the noncompetitive NMDA receptor antagonist properties of ibogaine. However, ibogaine, like another NMDA antagonist, MK-801, stimulates corticosterone release that has been shown to increase susceptibility to seizures.

The ECoG findings of a deficit in slow-wave brain activity after repeated exposure to cocaine confirmed observations in human subjects where addiction to cocaine was associated with deficits in delta power combined with reduced blood flow in the prefrontal cortex [20]. Our whole animal experimental approach may be useful in the study of drug dependence. The *in vivo* encephalography animal model offers opportunity to assess the baseline activity in the cortical areas before exposures to drugs of addiction occur. A similar approach would not be permitted in human subjects for ethical and legal reasons. 

This experimental setting presents a similar advantage in the study of GHB addiction. Analysis of spectral patterns obtained in this study provide a sensitive tool to investigate GHB interaction with neurotransmitters in the study of GHB addiction. Additionally, the EEG profiles obtained in the GHB experiment with behavioral effects showed similarity with absence epilepsy induced by GHB and induced by GABA agonists as reported earlier [21]. 

In summary, spectral analysis of the EEG signals combined with behavioral observations may prove to be a useful approach in studying chronic exposure to drugs of abuse and treatment of drug dependence. 

## Figures and Tables

**Fig. (1):**
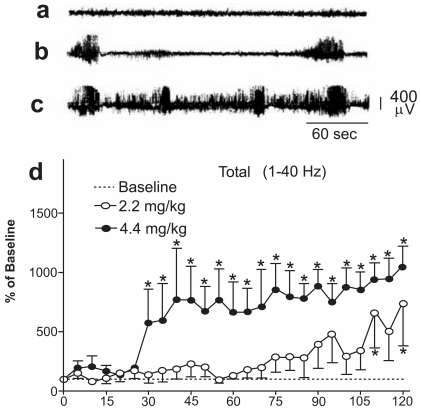
(a-c) Plots of the voltage as a function of time for the ECoG trace taken from the stripchart of a rat injected with DOM at 4.4 mg/kg. Baseline period (a), initial bursts of activity (b), increased rate of high frequency, high voltage ECoG bursts, and a lower voltage, lower frequency interictal spikes. (d) FFT of the 1-40 Hz frequency spectrum of rats injected with DOM at 2.2 mg/kg and 4.4 mg/kg. Power values were calculated as percent of the 30-min baseline power recorded after saline injection (assigned as value of 100% in each band). Experimental time in 15 min intervals is denoted on the X-axis. Mean ± SEM. n=5. *p<0.05 significantly different from baseline.

**Fig. (2):**
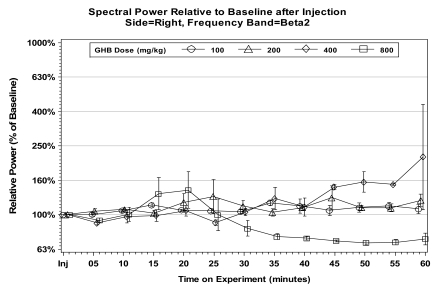
Effects produced by gamma-hydroxybutyrate injections at 100, 200, 400, and 800 mg/kg, i.p. on cortical EEG (ECoG) power spectra. Relative values calculated as percent of the 30-min baseline power assumed 100% in each band. Mean ± SEM.
